# Application of CO_2_-Switchable Oleic-Acid-Based Surfactant for Reducing Viscosity of Heavy Oil

**DOI:** 10.3390/molecules26206273

**Published:** 2021-10-16

**Authors:** Lulu Liu, Shuai He, Lu Tang, Shu Yang, Tao Ma, Xin Su

**Affiliations:** 1 State Key Laboratory of Shale Oil and Gas Enrichment Mechanisms and Effective Development, SINOPEC Exploration & Production Research Institute, Beijing 100083, China; llliu02@126.com (L.L.); yangshuwalcf.syky@sinopec.com (S.Y.); matao.syky@sinopec.com (T.M.); 2 Polymer Research Institute, State Key Laboratory of Polymer Materials Engineering, Sichuan University, Chengdu 610065, China; 3 College of Chemistry and Environmental Protection Engineering, Southwest Minzu University, Chengdu 610041, China; heshuaifish@126.com (S.H.); tanglu991@163.com (L.T.)

**Keywords:** CO_2_-switchable oligomeric surfactants, pseudo-tetrameric surfactants, viscosity reduction, demulsification

## Abstract

CO_2_-switchable oligomeric surfactants have good viscosity-reducing properties; however, the complex synthesis of surfactants limits their application. In this study, a CO_2_-switchable “pseudo”-tetrameric surfactant oleic acid (OA)/cyclic polyamine (cyclen) was prepared by simple mixing and subsequently used to reduce the viscosity of heavy oil. The surface activity of OA/cyclen was explored by a surface tensiometer and a potential for viscosity reduction was revealed. The CO_2_ switchability of OA/cyclen was investigated by alternately introducing CO_2_ and N_2_, and OA/cyclen was confirmed to exhibit a reversible CO_2_-switching performance. The emulsification and viscosity reduction analyses elucidated that a molar ratio of OA/cyclen of 4:1 formed the “pseudo”-tetrameric surfactants, and the emulsions of water and heavy oil with OA/cyclen have good stability and low viscosity and can be destabilized quickly by introducing CO_2_. The findings reported in this study reveal that it is feasible to prepare CO_2_-switchable pseudo-tetrameric surfactants with viscosity-reducing properties by simple mixing, thus providing a pathway for the emulsification and demulsification of heavy oil by using the CO_2_-switchable “pseudo”-oligomeric surfactants.

## 1. Introduction

It has been difficult to meet the current needs of industry and daily life with the output of conventional crude oil, and the exploitation and utilization of unconventional petroleum resources have been considered widely. Heavy oil is regarded as one of the most important unconventional petroleum resources due to its abundant reserves and relatively concentrated distribution [1]. However, its ultra-high viscosity restricts its exploitation, pipeline gathering and transportation [1]; thus, it is vital to identify efficient viscosity reduction methods for heavy oil. Generally, the methods useful for heavy oil viscosity reduction include both physical and chemical approaches [2]. The physical methods include heating [3] and thin oil mixing [4,5]. The mechanism of the chemical methods involves adding a catalyst [6], alkali [7,8], viscosity reduction agent or emulsifier [8] for reducing the viscosity. In the field of heavy oil gathering and transportation, a number of conventional emulsifiers can play an effective emulsifying role to decrease the viscosity of heavy oil; however, oil–water separation for heavy oil emulsions has always been an unresolved challenge. Lu et al. reviewed a number of advanced switchable molecules and materials for emulsification and viscosity reduction of crude oil [9]. Overall, it is of vital significance for the recovery and gathering of heavy oil to identify switchable emulsifiers, which can result in the swift and reversible emulsification and demulsification of the heavy oil.

The triggers of switchable emulsifiers generally include pH [10], light [11], temperature [12], redox environment [13] and CO_2_ [14]. However, the pH, light, temperature and redox environment suffer from significant usage limitations in oil fields, thus making them difficult to apply for viscosity reduction of heavy oil. CO_2_-switchable emulsifiers have attracted widespread attention due to their environmental friendliness, reusability and low cost [14,15,16,17]. CO_2_ is used as a stimulating trigger as CO_2_-switchable emulsifiers exhibit different properties in the absence or presence of CO_2_. In addition, the process of introducing and removing CO_2_ is simple, thus preventing the frequent replacement of the aqueous solution, which not only reduces the cost but also reduces environmental pollution [18,19,20]. Lu and co-workers prepared a series of CO_2_-responsive surfactants composed of fatty acids and EMA. The surface activity of the surfactants was observed to reach the desired state by controlling the CO_2_ incorporation so as to achieve the separation of the crude oil from the hosting solids and the harvest of the crude oil in the emulsion [21,22]. The findings obtained in these studies proved that the CO_2_-switchable surfactants have certain potential for the recovery and transportation of crude oil. Lu et al. synthesized switchable amphipathic molecules with switchable properties. Trigged by CO_2_, these molecules were used for the emulsification and demulsification of heavy oil, and the values of the critical micellar concentration (CMC) and lowest surface tension (γ_cmc_) were high; thus, the emulsification performance needed to be further improved [23]. Zhou et al. prepared a CO_2_-switchable polymer surfactant through the copolymerization of AM and DMAEMA, which was used to reduce the viscosity of heavy oil [1]. Huang and co-workers prepared a CO_2_-switchable oligomeric surfactant by using epoxidized soybean oil, which is proven to have an obvious viscosity reduction effect after sparging CO_2_ [24]. Although the properties of oligomeric surfactants were noted to be adjustable, they required efficient synthesis prior to application.

In this study, we prepared a CO_2_-switchable “pseudo”-tetrameric surfactant system by simply mixing oleic acid (OA) and cyclic polyamine (cyclen) with four amine groups. The surface tension and interfacial tension of the aqueous solution of OA/cyclen were studied by using surface and interfacial tensiometers. At a certain temperature, the heavy oil and the aqueous solution of OA/cyclen were proportioned to prepare a heavy oil emulsion with low viscosity and switchable stability; in addition, the demulsification effects of the OA/cyclen mixture were analyzed by bubbling CO_2_.

## 2. Results and Discussion

### 2.1. Properties of Heavy Oil

**Figure 1:**
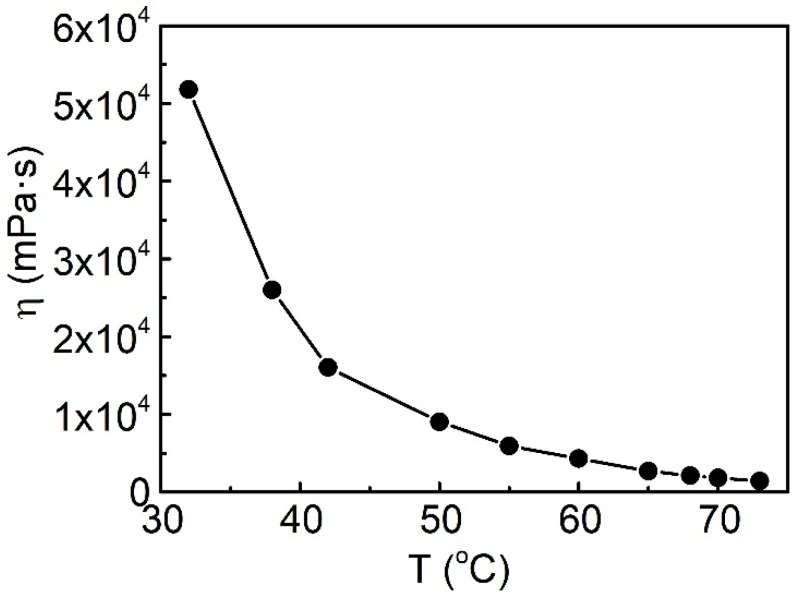
Viscosity–temperature plot of heavy oil from the Shengli oil field.

As observed from the viscosity–temperature plot in Figure 1, the viscosity of the heavy oil increased sharply on decreasing the temperature. At 50 °C, the viscosity of the heavy oil was determined to be 1.1 × 10^4^ mPa·s, attributed to the precipitation of the gelatin molecules from the heavy oil, thus forming a hydrogen-bonded network structure of a unique strength [25,26]. Figure 1 also demonstrates that the temperature had a significant effect on reducing the viscosity, thus enhancing the fluidity of the heavy oil. However, the method of enhancing the temperature to reduce the viscosity of heavy oil is expensive and consumes a significant amount of energy, which is not suitable for sustainable development of the oil industry [3]. As a result, an alternative method consuming a low amount of energy is required for reducing the viscosity of heavy oil.

### 2.2. Properties of Mixed OA/Cyclen as a CO_2_-Switchable Surfactant

**Scheme 1:**
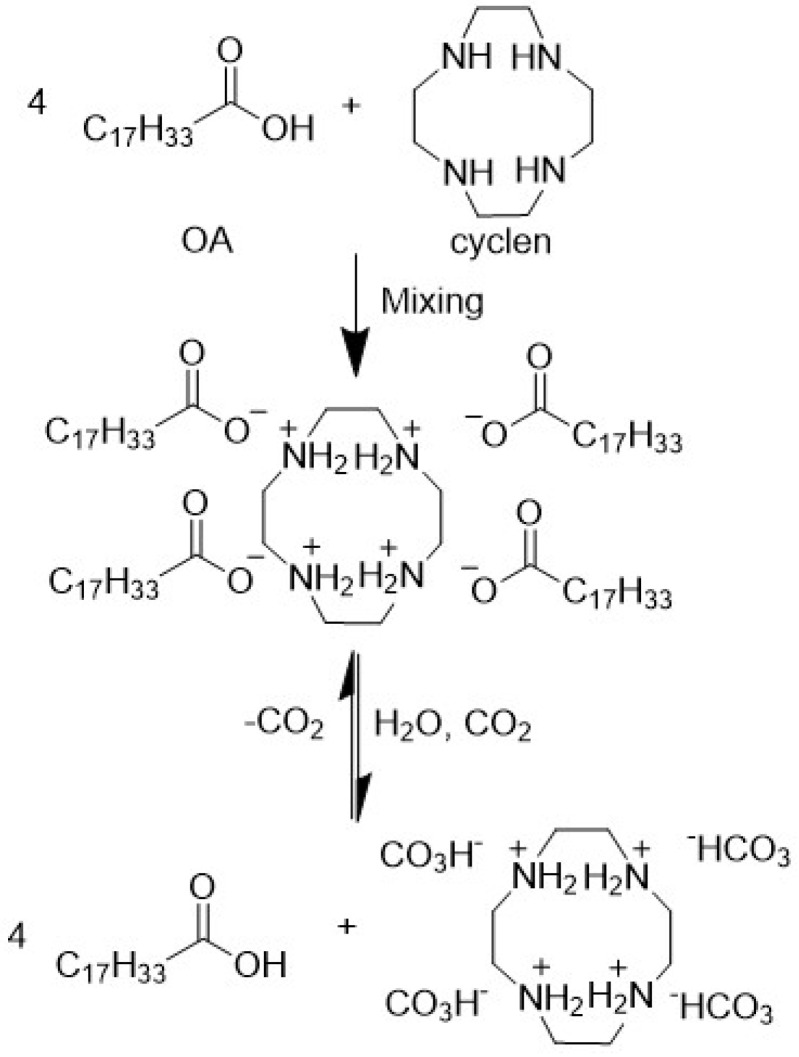
The CO_2_ switching mechanism of the mixed surfactant OA/cyclen.

The reaction between CO_2_ and amines leads to bicarbonate formation. Heating or bubbling an inert gas into the aqueous solution can reversibly transform the bicarbonate to the original amines state. Scheme 1 displays the switching reaction among cyclen, OA and CO_2_. Unlike the oligomeric surfactants formed by covalent bonds, without CO_2_, the mixed cyclen and OA form a cationic mixture, where OA possesses an anionic head group exhibiting anionic surfactant behavior. In the presence of CO_2_ dissolved in the aqueous solution, the anionic OA is protonated by CO_2_/H_2_O and turns neutral. Meanwhile, the cyclen cation and bicarbonate combine to form an ion pair. During this process, the cyclen cation does not demonstrate surfactant properties; thus, at this stage, no surfactant appears in the aqueous solution. Removing CO_2_ can lead to reversible deprotonation, and the OA/cyclen mixture forms an ion pair again.

#### 2.2.1. Surface Tension

**Figure 2:**
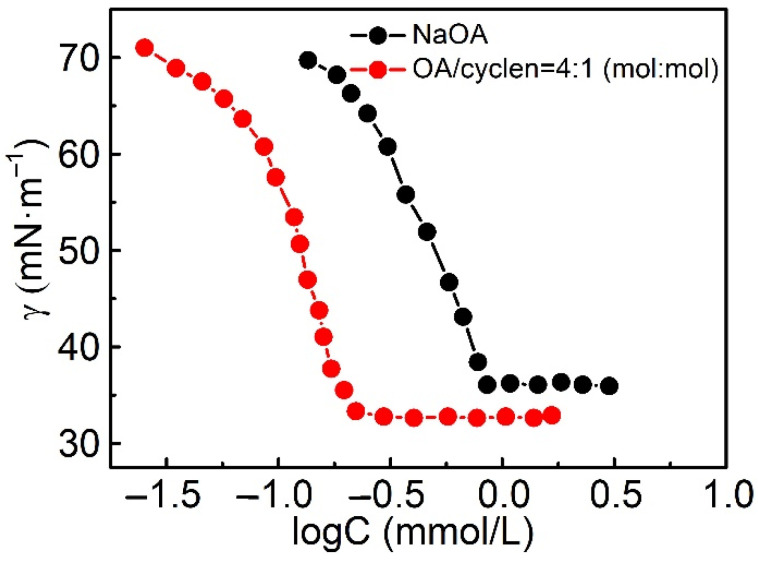
Plot exhibiting the variation in the surface tension with concentration for NaOA and OA/cyclen (mol/mol, 4:1) at 25 °C, where the concentration of OA/cyclen refers to the concentration of OA in OA/cyclen.

The CMC and γ_cmc_ of a surfactant can be obtained by measuring the surface tension as a function of the surfactant concentration. As shown in Figure 2, as the surfactant concentration increased, the surface tension was observed to gradually decrease. The observed surface tension reduction of the mixed system was not higher than that of the pure NaOA. As the concentration was increased further, the adsorption of the surfactant molecules on the air/water surface became saturated, and the surface tension did not change significantly anymore. As shown in Figure 2, the CMC value of the mixed surfactant was 0.30 mmol/L, which was less than that of the pure NaOA. This was attributed to the fact that cyclen in the mixture had multiple amino groups, which could form ion pairs with the OA molecules through non-covalent interactions, thus reducing the repulsion between the same charges. Table 1 shows the CMC and γ_cmc_ values of the different surfactants. As observed, OA/cyclen has lower CMC values compared to the CO_2_-switchable monomeric and dimeric surfactants, but similar to the value of the tetrameric surfactant, thus indicating its strong ability of micelle formation and micellar solubilization, and there is a stronger potential to reduce surface tension.

#### 2.2.2. Interfacial Tension

**Figure 3:**
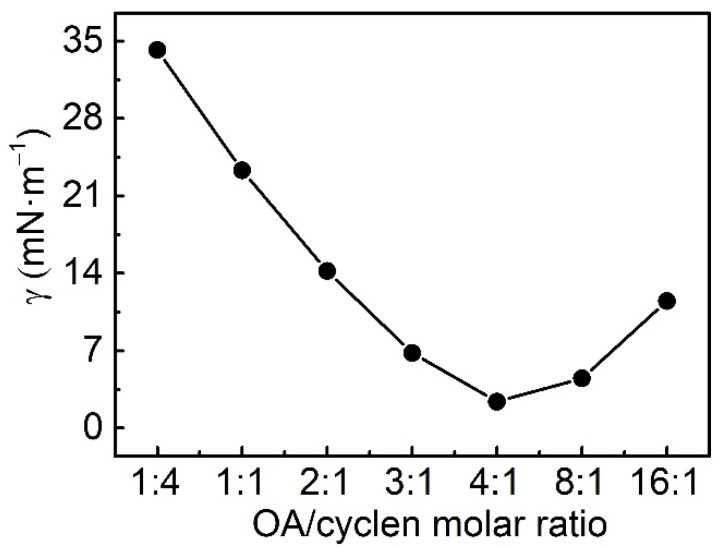
The variation in the interfacial tension with different molar ratios of OA and cyclen at 25 °C; the total concentration of OA/cyclen was fixed at 3.0 wt%.

As the surfactant is targeted for emulsifying heavy oil for reducing its viscosity, the interfacial tension of the surfactant represents a vital parameter, along with studying the influence of the molar ratio of OA and cyclen on the interfacial tension. As displayed in Figure 3, for the fixed total concentration of OA/cyclen of 3.0 wt%, increasing the proportion of OA first decreased the interfacial tension between the oil and water phases, followed by an increase. At a molar ratio of OA and cyclen of 4:1, the interfacial tension exhibited the lowest value of 2.4 mN/m. This was due to the fact that at a fixed total concentration of the surfactant, the ion pairs formed by OA and cyclen increased with the amount of OA, and the amount of OA anions acting as surfactants increased. The molar ratio of OA to cyclen of 4:1 formed the “pseudo”-tetrameric surfactants, and the amount of OA anions reached the maximum value. As the OA amount was continuously increased, the amount of cyclen decreased. Thus, the ion pairs formed by the two decreased, and the amount of OA anions acting as surfactants decreased. Therefore, at a molar ratio of OA and cyclen of 4:1, the interfacial tension exhibited the lowest value, and the low interfacial tension promoted the formation of the oil/water emulsion, thus achieving the emulsification and viscosity reduction of the heavy oil.

#### 2.2.3. CO_2_ Switching Behavior of OA/Cyclen Aqueous Solution

**Figure 4:**
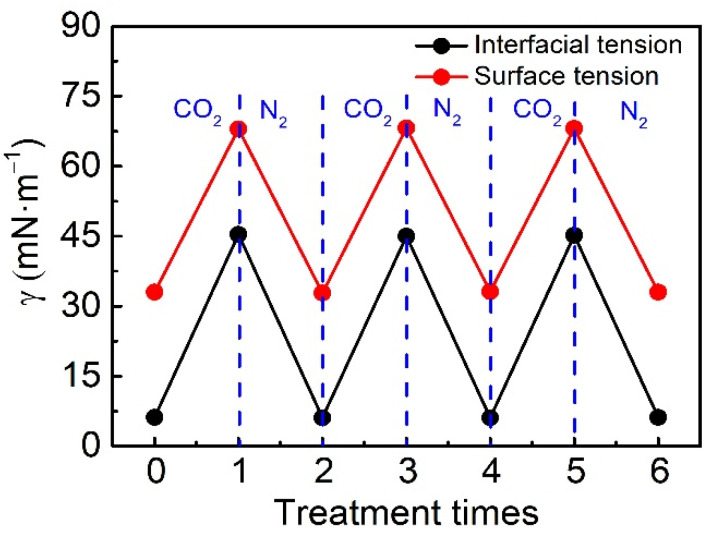
The variation in the surface tension and interfacial tension of the OA/cyclen (mol/mol, 4:1; (OA) = 0.5 mol/L) aqueous solution during alternate CO_2_/N_2_ bubbling at 25 °C.

The carboxyl groups of OA can be reversibly protonated before and after the implantation of CO_2_. Therefore, the reversibility of the surface tension and interfacial tension of the aqueous solutions with the mixed OA/cyclen surfactants was observed by sparging CO_2_/N_2_. Figure 4 shows the variation in the surface tension and interfacial tension after adding and removing CO_2_. The altering of the surface tension and interfacial tension resulted from the protonation/deprotonation of the carboxyl groups in OA. When the OA anions were protonated under CO_2_, the surface tension rose from 32 to 69 mN/m. As N_2_ was introduced and CO_2_ was removed, the surface tension of the aqueous solution returned to 32 mN/m. The same trend of reversible periodic change was observed in the case of interfacial tension, which altered between 5 and 47 mN/m. Three cycles were performed, and the CO_2_ switching behavior of the OA/cyclen aqueous solution was observed to be sustained.

### 2.3. Heavy Oil Viscosity Reduction

#### 2.3.1. Effect of Concentration

The concentration of the surfactant is critical for reducing the viscosity of the emulsion. An enhanced concentration strengthens the ability to reduce the interfacial tension of oil and water. Therefore, the optimal surfactant dosage of the mixed aqueous solution was determined. The molar ratio of OA and cyclen was set to 4:1, whereas the oil–water ratio was set as 7:3, and the emulsification temperature was adjusted to 50 °C.

**Figure 5:**
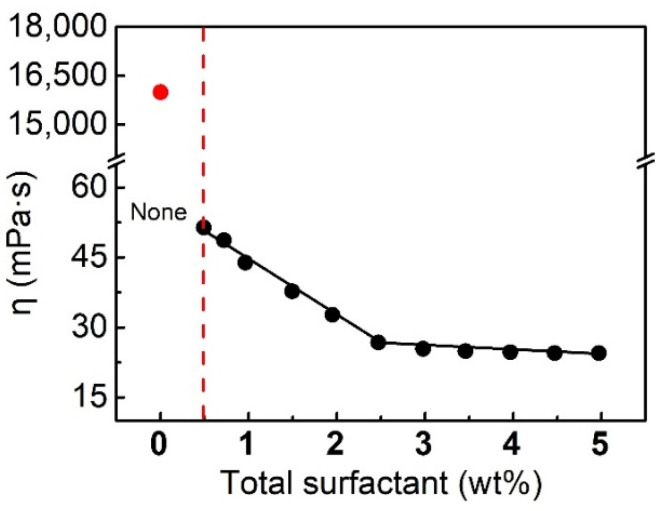
The influence of surfactant concentration on heavy oil emulsion viscosity at 50 °C. The molar ratio of OA and cyclen was set as 4:1, and the mass ratio of oil and OA/cyclen aqueous solution was set as 7:3.

The heavy oil viscosity after adding the surfactant was noted to be much lower than the original viscosity. As observed in Figure 5, when the surfactant concentration was higher than 0.5 wt%, the emulsion viscosity was 50 mPa·s, exhibiting suitability for pipeline gathering and transportation of the heavy oil. As the surfactant concentration was increased to 3.0 wt%, the viscosity reduction effect did not change significantly and reached a plateau value. At a concentration of less than 0.5 wt%, the emulsifying effect of the mixed aqueous solution of OA and cyclen was not strong enough, thus leading to almost no emulsification of the heavy oil. Therefore, the surfactant concentration of 3 wt% was selected for the emulsification and viscosity reduction of the heavy oil.

#### 2.3.2. Effect of Molar Ratio

**Figure 6:**
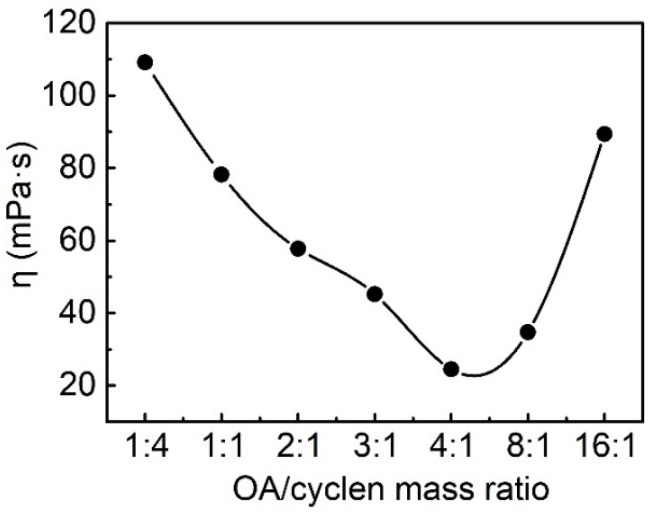
The influence of the molar ratio of OA and cyclen on heavy oil emulsion viscosity at 50 °C. The total concentration of OA and cyclen was 3.0 wt%, and the mass ratio of oil and surfactant OA/cyclen aqueous solution was set as 7:3.

To assess the effect of molar ratio, the total concentration of OA and cyclen was set as 3.0 wt%. As displayed in Figure 6, the molar ratio of OA and cyclen influenced the emulsion viscosity of the heavy oil from the Shengli oil field. Altering the molar ratio of OA and cyclen was observed to affect the heavy oil viscosity. For the molar ratio of OA and cyclen of 4:1, the emulsion viscosity exhibited the lowest value (28 mPa·s). This was because the mixture of OA and cyclen (molar ratio = 4:1) formed “pseudo”-tetrameric surfactants and resulted in the lowest interfacial tension (Figure 3), and the low interfacial tension easily formed an oil-in-water emulsion and reduced the viscosity of the heavy oil [1]. This consequently exhibited the optimal heavy-oil-emulsifying behavior. When the molar ratio of OA and cyclen was less than 1:1, the surfactant amount was small, and the heavy oil remained largely unemulsified.

#### 2.3.3. Effect of Temperature

**Figure 7:**
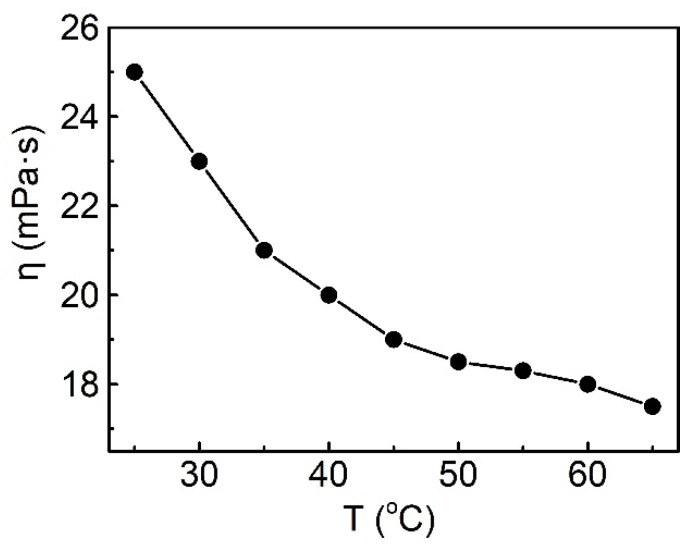
The effect of temperature on the viscosity reduction performance of OA and cyclen. The total concentration of OA and cyclen (molar ratio = 4:1) was 3.0 wt%, and the mass ratio of oil and OA/cyclen aqueous solution was set as 7:3.

As shown in Figure 7, temperature had a certain effect on the viscosity reduction performance; however, the effect was not too big. The heavy oil emulsions obtained in the temperature range of 25–65 °C were suitable for heavy oil production and gathering. As observed from Figure 7, the viscosity decreased by 30% in the investigated temperature range, which indicated that the mixture of OA and cyclen was slightly affected by temperature. Overall, the mixture still retained an optimal surface activity at high temperature for emulsifying the heavy oil to form an oil-in-water emulsion.

#### 2.3.4. Effect of Salinity

**Figure 8:**
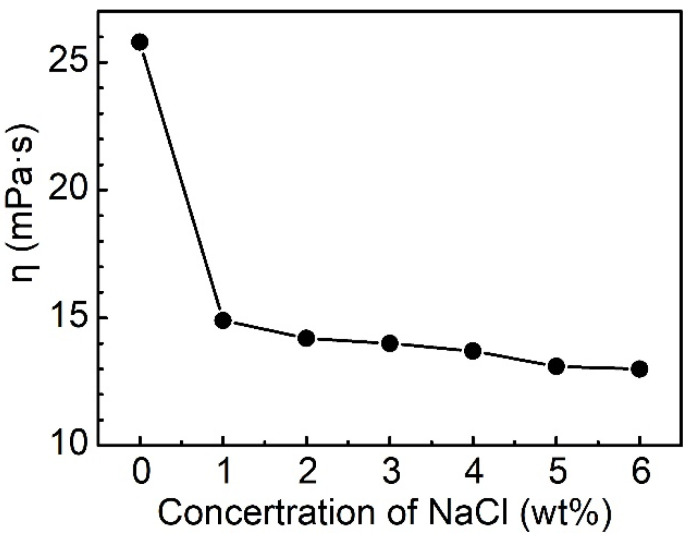
The effect of NaCl on the viscosity reduction performance of OA and cyclen. The total concentration of OA and cyclen (molar ratio = 4:1) was 3.0 wt%, and the mass ratio of oil and OA/cyclen aqueous solution was set as 7:3.

During the actual heavy oil production process, the salt concentration of the oil field is a critical factor. Thus, the influence of salinity on emulsification and viscosity reduction was studied. The total surfactant concentration was set as 3 wt%, the molar ratio of OA and cyclen was fixed at 4:1 and the mass ratio of oil and surfactant in the OA/cyclen aqueous solution was 7:3. The NaCl concentration of the aqueous solutions was varied, and the findings are presented in Figure 8. As the NaCl concentration was increased, the viscosity of the heavy oil emulsion gradually decreased. This was because the NaCl addition reduced the electrostatic repulsion between the polar heads of the surfactant molecules, and the surfactant was arranged more closely at the oil–water interface; the interfacial tension decreased, which reduced the viscosity of the heavy oil [29].

### 2.4. CO_2_ Demulsification of Viscosity Reduction System

**Figure 9:**
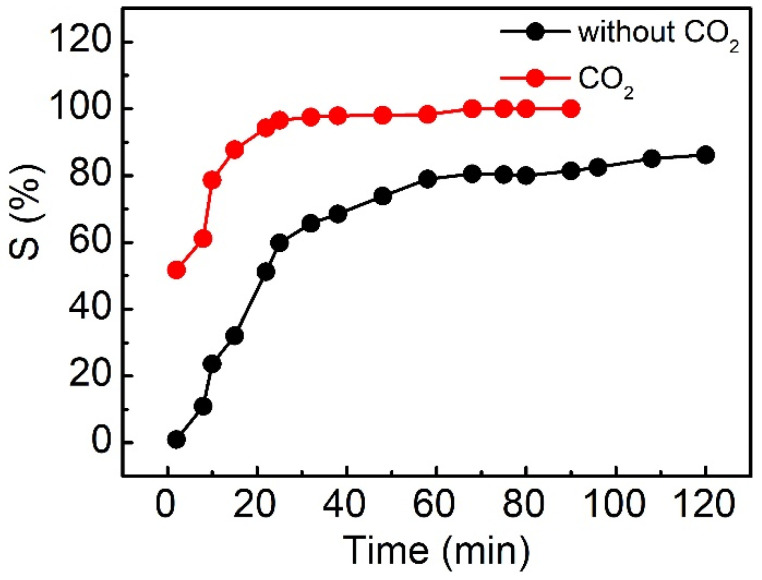
The effect of CO_2_ on the viscosity in the viscosity-reducing system. The total concentration of OA and cyclen (molar ratio = 4:1) was 3.0 wt%, and the mass ratio of oil and OA/cyclen aqueous solution was set as 7:3.

As observed, the heavy oil could be emulsified, and viscosity could be reduced by adding OA/cyclen. As demonstrated in Figure 9, in the absence of CO_2_, the heavy oil emulsion was naturally settled and dehydrated after keeping idle for a certain period of time, and the emulsified state was unstable. After adding CO_2_, the demulsification process was accelerated, and the dehydration rate exhibited an increase. It indicated that the heavy oil was produced in the form of an oil-in-water emulsion. After adding CO_2_, the pH value of the emulsion was switched, and the anionic groups in the oleic acid were protonated to reduce the overall surface activity of the emulsifier, thus destroying the protection of the inherent surface-active components at the oil–water interface. The presence of CO_2_ accelerated the demulsification and oil–water separation. After 20 min, the dehydration ratio (S%) was noted to reach 98.7%.

## 3. Experimental Procedures

### 3.1. Materials

Oleic acid (OA), sodium oleate (NaOA) and cyclen were purchased from Sigma-Aldrich. NaCl was obtained from Zhengzhou Paini Chemical Reagent Co., Ltd. CO_2_ (≥99.998%) and N_2_ (99.998%) were supplied by Xuyuan Chemical Industry Co., Ltd. (Chengdu, China) and were used as received. Ultrapure water with a conductivity of 18.25 μS·cm^−1^ was obtained by using an ultrapure water purification system (Chengdu Ultrapure Technology Co., Ltd., Chengdu, China). The heavy oil was supplied by the Shengli oil field, China.

### 3.2. Determination of Viscosity–Temperature Relation of Heavy Oil

The heavy oil was kept at a constant temperature for 6 h in a water bath for removing water and bubbles. Afterwards, the viscosity of the heavy oil was quickly measured as a function of temperature by using an MCR 302 rotational rheometer (Anton Paar, Graz, Austria) equipped with a Searle-type concentric cylinder geometry (CC27). The angular frequency was fixed at 7.0 s^−1^, and the corresponding viscosity–temperature relation was obtained.

### 3.3. Preparation of OA/cyclen Mixture

At room temperature, OA and cyclen were mixed in different molar ratios, followed by uniform stirring. Subsequently, the mixture was added to a saline solution or pure water to form a certain concentration of the surfactant solution.

### 3.4. Viscosity Reduction Experiments

To prepare a heavy oil emulsion, the heavy oil from the Shengli oil field was placed in a constant-temperature water bath for 1 h and stirred uniformly with a magnetic stirrer to remove the bubbles and free excess water. The initial viscosity of the heavy oil at a constant temperature was determined using the MCR 302 rotational rheometer. Subsequently, the aqueous solution of OA/cyclen and the heavy oil were added in a beaker at a mass ratio of 7:3 to obtain an emulsion. The emulsion was maintained at a constant temperature, with magnetic stirring at 1200 rpm for 15 min so as to prepare a heavy oil emulsion. Meanwhile, the viscosity of the heavy oil emulsion was analyzed using the MCR 302 rotational rheometer.

To measure the emulsion stability, a specific amount of the aqueous solution of OA/cyclen was taken in a graduated cylinder. The solution volume was measured, followed by addition of the heavy oil to adjust the mass ratio of the heavy oil and aqueous solution to 7:3. The mixture was thoroughly stirred to obtain an emulsion. The graduated cylinder was placed at room temperature for 2 h to explore the clear oil–water separation layering phenomenon, followed by recording of the water volume.

To evaluate the demulsification effect, CO_2_ was bubbled into the graduated cylinder with the freshly prepared emulsion to analyze the oil–water separation, followed by recording of the water volume. The dehydration ratio (S%) was calculated based on the following equation [23]:

(1) $$S\%=\frac{Dehydration\;volume\;of\;water}{Total\;volume\;of\;water\;in\;emulsion}\times 100\%$$

### 3.5. Surface Tension Analysis

The surface tension of the OS solutions was determined using a K100 tensiometer (Kruss, Germany) at 25 °C. Each sample was measured thrice to determine an average value. The critical micelle concentration (CMC) of the aqueous solutions was determined using the plot of the surface tension and logarithmic concentration.

### 3.6. Interfacial Tension Analysis

A TX500C interface tensiometer was used to measure the interfacial tension by stretching the oil droplets. First, the tube was filled with the aqueous solution. Afterwards, a drop of oil was injected into the aqueous solution with a syringe to obtain the oil–water interface. Finally, the oil droplet was stretched and deformed in the interface tensiometer. After the shape of the oil droplet became stable, the interfacial tension data were recorded.

## 4. Conclusions

In this study, a CO_2_-switchable “pseudo”-tetrameric OA/cyclen surfactant was prepared using simple mixing. The aqueous solution with OA/cyclen exhibited lower values of CMC, γ_cmc_ and interfacial tension as compared to some CO_2_-switchable oligomeric surfactants, thus exhibiting a significant potential to reduce heavy oil viscosity. By varying the experimental conditions, it was observed that a total surfactant concentration of 3 wt% and a 4:1 molar ratio of OA to cyclen in the surfactant aqueous solution formed “pseudo”-tetrameric surfactants, exhibiting the optimal emulsification and viscosity reduction effect. The presence of CO_2_ could accelerate the demulsification and oil–water separation of heavy oil emulsions. This study provides a strategy for designing CO_2_-switchable “pseudo”-tetrameric surfactants with a superior surface activity by simple mixing and sheds light on the emulsification and demulsification of heavy oil by using CO_2_-switchable “pseudo”-oligomeric surfactants.

## Figures and Tables

**Table 1:** Comparison of the surface properties of different CO_2_-switchable surfactants.

Molecular Structure of Surfactant	Type	CMC (mM)	γ_cmc_ (mN·m^−1^)	Source of Data
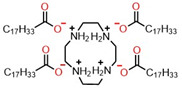	Pseudo-tetrameric	0.30	32.75	This work
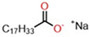	Monomeric	0.86	36.07	This work
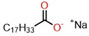	Monomeric	1.20	25.70	Ref. [27]
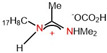	Monomeric	9.46	45.05	Ref. [23]
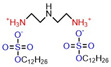	Dimeric	0.67	30.25	Ref. [28]
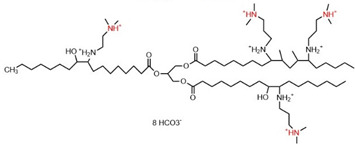	Oligomeric	0.20	35.80	Ref. [24]

## Sample Availability

Samples of the compounds mentioned in the paper, including sodium oleate, cyclic polyamine and heavy oil are available from the authors.
